# COMMUNICATION TRAINING FOR MEDICAL PROFESSIONALS: PROOF OF CONCEPT

**DOI:** 10.1093/geroni/igae098.2695

**Published:** 2024-12-31

**Authors:** Nancy Brown, Vicki De Klerk-Rubin

**Affiliations:** Validation Training Institute, Jerusalem, Yerushalayim, Israel; Validation Training Institute, Springfield, Oregon, United States

## Abstract

Despite the prevalence of patients living with dementia, physicians and medical students in particular, have received little or no dementia-specific training to help them communicate effectively with this population. Connecting and communicating with persons living cognitive change requires specific skills. These skills are taught in the Validation for Physicians and Medical Provider training, based upon the evidence-based Validation method by Naomi Feil. Developed with physicians and pedagogical expert in online learning, the training was designed for healthcare professionals, specifically doctors, with quality of care for patients and their carers in mind. The training comprised of a variety of teaching methods including: online on-demand videos, quizzes, case studies, and live webinars for each of the five blocks. The pilot program included physicians and other healthcare professionals. Pre and Post surveys consisted of a knowledge scale and SRDB (self-efficacy) ranked on a Likert scale of 1 to 5 and analyzed using SPSS. Post-training individual interviews concluded the beta-testing. Thirty professionals enrolled in the training; eight participants completed every aspect of the beta-testing. Despite the small sample size, there were trends approaching significance in knowledge and self-efficacy. Participants highly valued the contents of the training as well as the pedagogy. Technical problems were the main reason for attrition. Overall, the Validation method was found useful in practice, improved confidence, knowledge, and communication behaviors. Improvements are needed in the choices for validated measures and the online platform. “Validation has made me a better doctor. It has changed my perspective on aging” (participant).

